# Assessing the Value of Imaging Data in Machine Learning Models to Predict Patient-Reported Outcome Measures in Knee Osteoarthritis Patients

**DOI:** 10.3390/bioengineering11080824

**Published:** 2024-08-12

**Authors:** Abhinav Nair, M. Abdulhadi Alagha, Justin Cobb, Gareth Jones

**Affiliations:** 1 MSk Lab, Department of Surgery and Cancer, Faculty of Medicine, Imperial College London, London, UK; 2 Data Science Institute, London School of Economics and Political Science, London, UK

**Keywords:** knee osteoarthritis, WOMAC, machine learning, imaging, radiograph, MRI

## Abstract

Knee osteoarthritis (OA) affects over 650 million patients worldwide. Total knee replacement is aimed at end-stage OA to relieve symptoms of pain, stiffness and reduced mobility. However, the role of imaging modalities in monitoring symptomatic disease progression remains unclear. This study aimed to compare machine learning (ML) models, with and without imaging features, in predicting the two-year Western Ontario and McMaster Universities Arthritis Index (WOMAC) score for knee OA patients. We included 2408 patients from the Osteoarthritis Initiative (OAI) database, with 629 patients from the Multicenter Osteoarthritis Study (MOST) database. The clinical dataset included 18 clinical features, while the imaging dataset contained an additional 10 imaging features. Minimal Clinically Important Difference (MCID) was set to 24, reflecting meaningful physical impairment. Clinical and imaging dataset models produced similar area under curve (AUC) scores, highlighting low differences in performance AUC < 0.025). For both clinical and imaging datasets, Gradient Boosting Machine (GBM) models performed the best in the external validation, with a clinically acceptable AUC of 0.734 (95% CI 0.687–0.781) and 0.747 (95% CI 0.701–0.792), respectively. The five features identified included educational background, family history of osteoarthritis, co-morbidities, use of osteoporosis medications and previous knee procedures. This is the first study to demonstrate that ML models achieve comparable performance with and without imaging features.

## 1. Introduction

Osteoarthritis (OA) is the primary contributor to disability and chronic pain among patients over 60 years of age [1]. Knee OA is estimated to affect 654 million people worldwide, with pain and joint stiffness significantly impacting daily activities, quality of life and emotional well-being, particularly among the ageing population [2,3]. Patient-reported outcome measures (PROMs), such as the Oxford Knee Score (OKS) and the Western Ontario and McMaster Universities Arthritis Index (WOMAC), are widely utilised to evaluate symptoms in knee osteoarthritis [4]. WOMAC is a reliable multi-dimensional health status assessment tool that is considered the gold standard in evaluating OA severity and monitoring disease progression [5,6,7]. The added advantage of WOMAC over other PROMs is that it gives a more comprehensive evaluation of pain, stiffness and physical function [5,6]. It may also aid in decision-making around the timing of total knee arthroplasty (TKA) [8,9]. Recent studies have highlighted the need for reliable prediction of future PROMs to enhance the shared decision-making process with patients regarding their long-term treatment options [10].

Imaging modalities demonstrated increased diagnostic sensitivity for symptomatic individuals with knee OA; nevertheless, their specific advantage in guiding clinical decision-making regarding management options remains uncertain [11,12]. Erlangga et al.’s systematic review found limited or inconclusive associations between magnetic resonance imaging (MRI) findings and OA-related knee pain across 22 studies [13]. However, moderate levels of evidence were found for imaging features such as bone marrow lesions and synovitis being an indication of knee pain in such patients [13]. Moreover, other studies reported certain MRI features identified in symptomatic patients that were also evident in asymptomatic individuals [11,14].

The increased use of imaging has led to a significant rise in healthcare expenditure around the world. Recent figures show the costs of unnecessary knee MRIs by a single consultant in the United Kingdom to be over GBP 13,000 per year [15]. Nationally, a study from Norway estimated that unnecessary knee MRIs cost around EUR 6.7–EUR 9.8 million every year [16]. Similarly, for plain radiographs, a study by Ashikyan et al. analysing the medical records of 500 knee OA patients in the United States found that over 15% of the six-month follow-up radiographs performed were deemed non-essential to patient care, amounting to USD 10,800 per year [17]. The financial impact and radiation risks add to the need for further research on the role of imaging in forecasting meaningful clinical changes in knee OA patients [18,19].

Whilst traditional statistical models are inherently constrained by their linearity assumptions and limited capacity to handle complex datasets, machine learning (ML) algorithms exhibit superior predictive power and feature engineering advantages [20,21,22]. In knee OA, previous ML studies have predominately focused on predicting objective outcomes such as joint space narrowing and the need for TKR, overlooking other vital domains for these patients such as stiffness and functional ability [23,24]. Two studies have compared models based on clinical data alone, with those based on MRI data, in predicting the necessity for a TKA, concluding that incorporating MRI scans was of no additional value [25,26,27]. However, the role of imaging in predicting symptoms, which are the main drivers for patients’ treatment choices, remains unexplored, as does the need for robust external validation of such models [28,29].

This study aimed to develop and externally validate ML models, and comparatively evaluate their performance with and without imaging features, to forecast the 2-year WOMAC score of patients with knee OA. Secondary objectives included identifying the most influential features that contribute to the predictive ability of the top-performing model. We hypothesised that machine learning models lacking imaging features would demonstrate comparable performance, as evaluated by the Area Under Curve metric, to models incorporating imaging features in predicting the 2-year WOMAC scores.

## 2. Materials and Methods

### 2.1. Ethics Considerations

No ethical approval was required for this study owing to the open access nature of the OAI and MOST databases. Ethical approval and informed consent for collecting data about participants were obtained by the OAI and MOST datasets. The OAI dataset is hosted by the Osteoarthritis Initiative Data Coordinating Center (OAI DCC) at the University of California, San Francisco (UCSF), and is available through the National Institute of Health (NIH) NIAMS repository: https://nda.nih.gov/oai (accessed on 5 March 2021). The MOST data are accessible through the MOST Online Data Repository and supported by the NIH NIAMS: https://most.ucsf.edu/ (accessed on 5 March 2021).

### 2.2. Data Source

This study used the Osteoarthritis Initiative (OAI) database (https://nda.nih.gov/oai/, accessed on 5 March 2021) to train and internally validate the ML models, and the Multicenter Osteoarthritis Study (MOST) database (https://most.ucsf.edu, accessed on 5 March 2021) to externally validate its performance. Both OAI and MOST databases are multi-centre longitudinal prospective studies assessing men and women in the United States with, or at high risk of, symptomatic knee OA. OAI enrolled 4796 subjects aged between 45 and 79 years from February 2004 to May 2006, while MOST enrolled 3026 subjects aged between 50 and 79 years from April 2003 to April 2005. Both databases are publicly available, with the study design and protocol approved by local institutional boards of participating centres and informed consent obtained from all participants [30,31]. Imaging features used from OAI and MOST databases were read by trained assessors, blinded to patient details and clinical status. MRI scans were read using the Whole-Organ Magnetic Resonance Imaging Score (WORMS) scoring system.

### 2.3. Outcome Measure

Our primary outcome was the binary 2-year WOMAC change (improvement/no improvement), which comprises three domains including knee pain, stiffness and functional limitations, as reported by the participants. The WOMAC questionnaire consisted of a total of 24 items, with each question being scored between 0 (None) and 1–4 (Mild–Extreme) points. A threshold score of 24 was selected based on the minimal clinically important difference (MCID) from previous studies that suggested meaningful symptomatic physical impairment in patients [32,33,34]. In other words, a total WOMAC score of 24 and above was categorised as clinically symptomatic (positive class), while a score below 24 (negative class) was considered less significant.

### 2.4. Feature Selection and Data Pre-Processing

In total, 1187 and 553 features were analysed using the OAI and MOST databases, respectively. To enable external validation, only variables present in both OAI and MOST databases with ≥60% completeness were included in this study.

In an attempt to generate explainable ML models, the features were systematically transformed into meaningful categorical comparisons in both databases. This process involved converting continuous variables, such as blood pressure, into discrete stages of hypertension, as per clinical guidelines [35]. Inter-related features such as pain medications were combined into a single variable based on the WHO analgesic ladder (no analgesia, non-steroidal anti-inflammatory drugs, narcotics) to prevent the dilution of features and improve model performance [36,37]. Features exhibiting high collinearity, such as the type of surgery performed, were excluded to reduce to mitigate redundancy. Table 1 shows the final set of features selected for model training, with additional information on the classification levels for each feature presented in Appendix A.

Two separate datasets were created to evaluate the role of imaging features in predicting meaningful clinical changes in 2-year WOMAC scores. As shown in Table 1, the clinical dataset contained the selected clinical features but not the radiographic and MRI variables. The imaging dataset contained the features in the previous dataset in addition to the radiographic and MRI variables. Subjects (n = 608) that had an MRI performed in both legs or more than once at baseline were recorded and analysed as separate observations.

**Figure 1:**
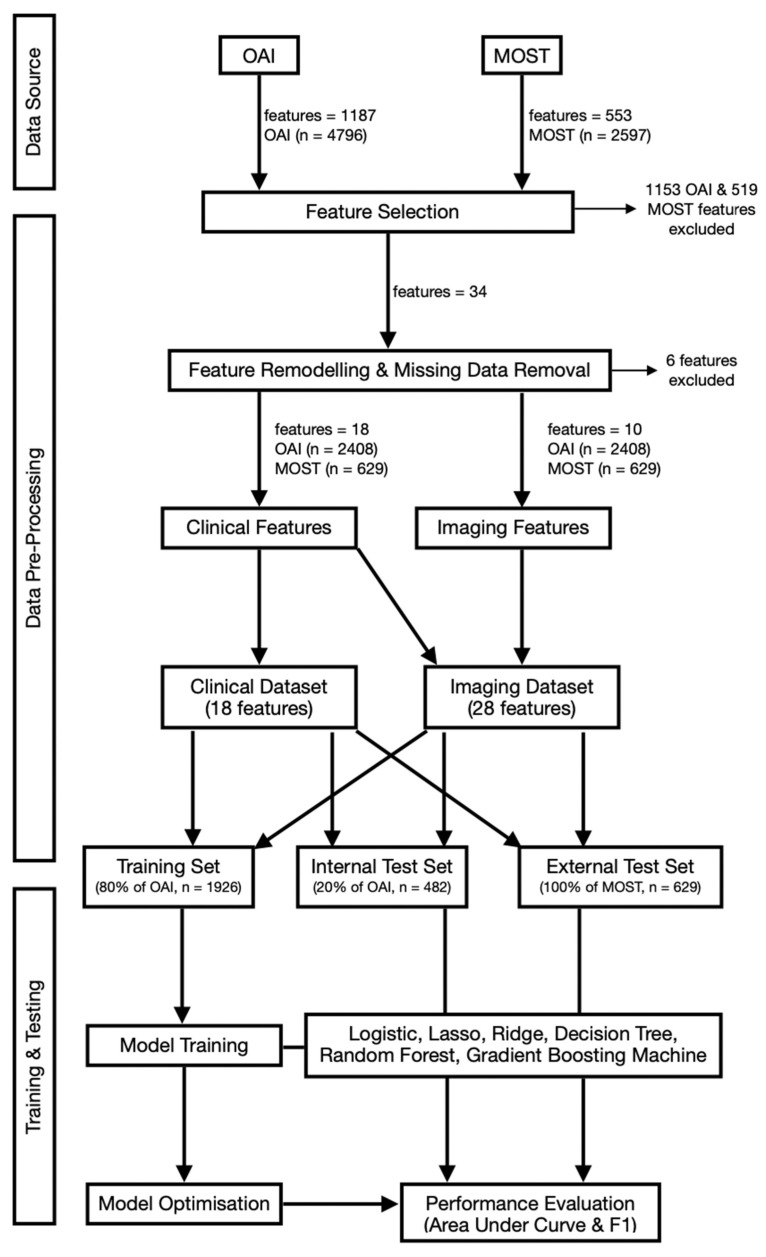
Flowchart summarising the methodology from data extraction to model training and testing for Osteoarthritis Initiative (OAI) and Multicenter Osteoarthritis Study (MOST) databases.

Patients with missing data in any of the final features were removed from this study. In the OAI database, both clinical and imaging datasets had their observations split into 80% training and 20% internal validation sets. The MOST database was later used to allow for an unbiased external validation. Figure 1 shows a summary of the pre-processing, training and testing stages.

### 2.5. Model Development, Training and Validation

Five linear and tree-based classification machine learning algorithms were developed, namely, Least Absolute Shrinkage (LASSO) Regression, Ridge Regression, Decision Tree (DT), Random Forest (RF) and Gradient Boosting Machine (GBM), to minimise variance and bias, and then compared to traditional multivariate Logistic Regression (LR), to predict the binary 2-year WOMAC change (improvement/no improvement) [38,39,40,41,42]. This was followed by hyperparameter tuning via 10-fold cross-validation in the LASSO, Ridge, RF and GBM models to reduce over-fitting in the training set [43]. No tuning was required for the base models using linear (LR) and tree-based (DT) algorithms. The models were then tested on the previously unused MOST database for external validation. Model performance was assessed in terms of the gold-standard area under the receiver operating characteristic curve (AUC) [44]. To account for the class imbalance between positive and negative cases in the outcome measure, the F1 score was computed [45]. Given that the F1 score is dependent on the decision threshold to convert outcome probabilities into discrete classes, its decision thresholds were optimised to maximise the score and achieve the highest positive predictive ability.

### 2.6. Statistical Analysis and Feature Importance

Descriptive statistics, including mean and percentage, were used to describe the rates of changes in the 2-year WOMAC score across the OAI and MOST databases. All mathematical modelling was carried out using R statistical computing environment version 4.3.0 (R: A language and environment for statistical computing). R packages ‘survival’ (version 3.6-4), ‘gbm’ (version 2.1.8), ‘glmnet’ (version 4.1-4), ‘tree’ (version 1.0-43), ‘rpart’ (version 4.1.16), and ‘randomForest’ (version 4.7-1.1) were used for survival analysis. Further details of software packages are provided in Appendix A. Two-tailed Wilcoxon Signed-Rank test was used to assess the difference in non-parametric AUC scores of imaging and clinical dataset models. However, caution is advised when interpreting the results since our findings are not subject to inferential testing and do not establish statistical significance.

## 3. Results

### 3.1. Data Distribution

The final dataset included 2408 and 629 observations from the OAI and MOST databases, respectively. Descriptive statistics related to patient demographics, clinical data and imaging features for OAI and MOST databases are presented in Table 2.

As highlighted in Table 2, the majority of patients were from White or Caucasian ethnic backgrounds, amounting to approximately 84.3–89.5% of observations. Variances between the OAI and MOST databases were notable in the analgesic medication, Kellgren–Lawrence (KL) grade and patellofemoral joint cartilage morphology. Nevertheless, a comparable proportion of patients exhibited normal WOMAC baseline scores in both databases (OAI: 73.7%, MOST: 73.1%).

### 3.2. Model Performance

The OAI database was split into 80% (n = 1926) training and 20% (n = 482) internal validation cohorts, whilst 100% of the MOST database (n = 629) was used for external validation. Table 3 shows the AUC values for the six models in the training and internal validation sets.

ML models in the datasets from OAI had AUC score ranges of 0.628–0.820 in the training and 0.630–0.786 in the internal validation sets. These scores consistently surpassed the clinically acceptable threshold of AUC > 0.70, with the exception of the Decision Tree (DT) algorithm (AUC = 0.62–0.66). Across both clinical and imaging datasets, the Random Forest (RF) algorithm was the best-performing model in both training and internal test sets (AUC = 0.77, AUC = 0.78, respectively). There was comparable model performance, in terms of AUC values, between the clinical and imaging datasets, with a marginal difference of 0.025.

**Figure 2:**
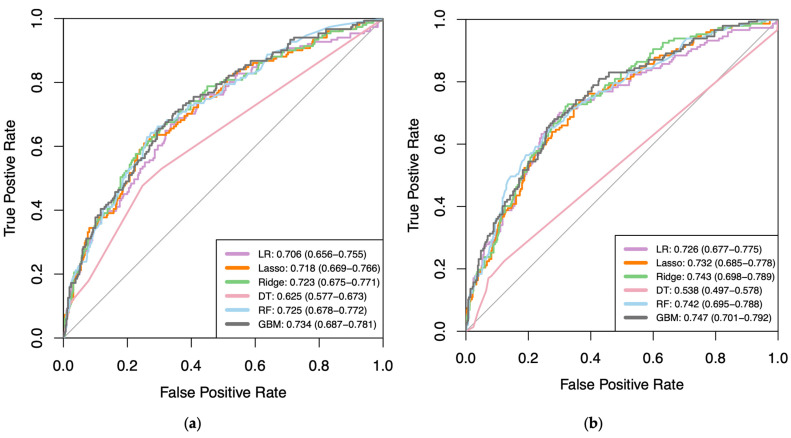
Receiver operating characteristic (ROC) curves showing Area Under Curve (AUC) scores (with 95% confidence intervals) of all six (**a**) clinical and (**b**) imaging machine learning algorithms at external validation from Multicenter Osteoarthritis Study (MOST). Thin black line represents performance of a random classifier (AUC = 0.500). All values shown to three significant figures. LR, Logistic Regression; DT, Decision Tree; RF, Random Forest; GBM, Gradient Boosting Machine.

Figure 2 highlights the AUC receiver operating characteristic curves (ROC) for the six models in the external validation MOST cohort. GBM models performed with the highest AUC in the external test for clinical (AUC = 0.734) and imaging datasets (AUC = 0.747). Due to the low performances (AUC < 0.7) in both internal and external tests, DT was excluded from further analysis. Besides the DT algorithm, which had the lowest predictive ability (AUC < 0.70), variations in AUC scores between clinical and imaging datasets were minimal (<0.02) across all other models, with no statistical differences (*p* > 0.05). The ROC curves for training and internal validation sets are provided in Appendix B.

Excluding the DT model, the F1 score, a relative measure of a model’s ability to identify true positive classes, exceeded 0.5 for all models in both clinical and imaging datasets (Table 4). Random Forest (RF) and Gradient Boosting Machine (GBM) achieved the highest F1 scores in internal (F1 = 0.617) and external (F1 = 0.548) tests, respectively. Similar to AUC values, there was no significant variation in F1 scores between clinical and imaging datasets (<0.03). Further information on the change in AUC and F1 scores between internal and external tests is provided in Appendix B.

### 3.3. Feature Importance

The GBM model was the best-performing model across both clinical and imaging datasets, as evaluated by the AUC score. The top five most influential factors that affected the predictive ability of GBM models are given in Table 5.

The patients’ educational background exerted the most substantial influence on the predictive ability of the GBM model in the clinical dataset, accounting for 22% of feature importance, and ranked second with approximately 8% in the imaging dataset (Table 5). In the clinical dataset, this was followed by factors such as osteoarthritis history, co-morbidities, medicated osteoporosis and a previous knee operation. Conversely, in the imaging dataset, the most influential feature was KL grade (~10%). Other contributing features in the imaging model included the 20 m walk test, joint space narrowing (JSN) in the medial compartment, and the use of analgesics.

## 4. Discussion

This study aimed to evaluate whether machine learning models can attain comparable performance in predicting a binary outcome in the 2-year WOMAC score in patients with knee OA, and compare that to traditional logistic regression, irrespective of the inclusion of MRI and radiographic features, using the OAI and MOST databases. Our findings highlighted comparable predictive capabilities, with minimal differences, of less than 0.025 in the area under the curve (AUC) values, whether the models incorporated imaging features or not. The GBM algorithm demonstrated the highest AUC and F1 scores at external validation, achieving similarly acceptable scores for imaging dataset models, and outperforming the logistic regression model.

This study adopted an ML approach and identified that the best-performing models were tree-based algorithms (RF, GBM). Previously, Bastick et al. utilised LR algorithms to detect pain trajectories and predict patient symptoms from their clinical data [46]. However, our findings align with previous studies underscoring the superior predictive performance of tree-based algorithms in capturing non-linear relationships when compared to traditional statistical methods [47,48]. Importantly, the GBM and the RF models consistently achieved the highest AUC for both clinical and imaging datasets, demonstrating clinically acceptable scores upon external validation, a facet not extensively addressed in prior research [49].

In terms of feature importance, the GBM algorithm in our study alluded to educational background as the most important predictive driver in both clinical and imaging datasets. A previous cross-sectional study analysed the relationship between clinical features and knee OA and reported educational background to have the highest significant negative correlation with a patient’s current WOMAC score [50]. Their findings highlighted that the education status of patients with knee OA was likely to impact their future functional and QoL outcomes [50]. This may be explained by the hidden confounding factors within educational background such as income and type of employment that affect knee OA progression [51]. Additionally, low education levels may contribute to a lack of knowledge and awareness regarding lifestyle modifications for managing OA [52]. However, in our study, it is important to consider this finding with caution due to the lack of inference testing, inherent limitations of the OAI and MOST databases, and ML algorithmic bias [53,54,55]. Future prospective studies are needed to evaluate this causal inference.

In the imaging dataset, the Kellgren–Lawrence (KL) grade from radiographs exerted the most substantial overall influence on the imaging dataset’s GBM model, while no MRI features were identified as highly influential in enhancing the predictive ability of the machine learning model. A previous study that used the MOST database included 696 observations and showed a higher occurrence of symptomatic knee pain in KL grades 1–4 (Odds Ratio: 1.5, 3.9, 9.0, 151; respectively) as compared to KL grade 0 [56]. Whilst our study favoured explainable machine learning models over the black box approach posed by DL, another study using 9348 observations from the OAI database showed that radiographs analysed through deep learning (DL) alone (AUC = 0.770) were able to predict the symptomatic progression of knee OA better (*p* < 0.001) than the clinical features (AUC = 0.692) [57]. While it has been shown that MRI features are associated with patients experiencing knee pain, individual features were ineffective in discriminating between painful and non-painful knees [58]. This suggests that more studies are required to evaluate DL approaches in analysing MRI features for symptom prediction.

Interestingly, Ashinsky et al. used only MRI features from the OAI database to predict a 3-year change in WOMAC score, achieving a 75% classification accuracy [59]. Their findings reported cartilage thickness at the central portion of the medial femoral condyle to be the feature that most affected symptomatic OA progression [59]. Whilst cartilage thickness was not shown to be a key feature in our study, this is likely due to the greater influence of clinical factors in predicting 2-year WOMAC. Schiratti et al., who combined MRI data with clinical features from the OAI database to predict patients’ 1-year WOMAC pain scores, achieved a lower AUC score of 0.724 than the GBM in our study (AUC = 0.747) [60]. This may be because they utilised raw MRI images, which can add unnecessary noise to the dataset, which is counterproductive in improving model performance [61]. Their study also suggested that intra-articular space has the highest contribution in predicting the patient’s pain, which was not recorded in the datasets of our study [60,62]. Therefore, this may be a significant feature to obtain in future studies to boost the model’s predictive ability.

This study employed two of the largest available osteoarthritis databases (OAI and MOST) to test and externally validate our ML models. Moreover, the input features curated were commonly assessed and recorded for all OA patients, increasing the explainability and application of our models in the real world. However, there are limitations to report.

Firstly, the databases drew subjects exclusively from the United States, resulting in limited diversity in ethnic background, educational status, and socioeconomic factors. Consequently, the generalisability of our models to international contexts with diverse backgrounds might be constrained. Additionally, while the images underwent evaluation by individuals following a standardised scoring system, the inherent subjectivity of this process, as documented in previous research, is a potential limitation [63]. A comparative analysis with DL models utilising raw images and continuous data could offer an alternative perspective on their role in predicting outcome scores. Lastly, the models were developed using an imbalanced class dataset, with a minority (26.5%) having a high WOMAC score. This presents a challenge for machine learning algorithms and potentially diminishes their performance. Future research could address this issue through under-sampling methods, such as the K-nearest neighbour algorithm, to rebalance the data [64].

This study underscores the effectiveness of machine learning (ML) models in predicting knee osteoarthritis (OA) severity, as measured by changes in the 2-year WOMAC score, using routinely recorded clinical data without the need for additional imaging. The practical application of predicting WOMAC scores holds promise for clinicians to evaluate the progression of health-related quality of life in knee OA from an early stage, enhancing the shared decision-making process and tailoring patient management strategies. Early, smaller interventions, such as focal resurfacing or unicompartmental knee arthroplasty (UKA), for patients at high risk of developing severe symptoms could potentially enhance their long-term QoL [9].

Future studies are needed to evaluate ML’s ability to forecast even longer-term WOMAC scores. While our study focused on symptomatic patients with existing knee OA, the evaluation of WOMAC scores in asymptomatic patients might be helpful in aiding the early decision-making process. Finally, the use of ML in predicting other PROMs that assess QoL, such as SF-36, would enable clinicians to adopt a more holistic approach to patient care in the future.

## 5. Conclusions

This study demonstrated that machine learning (ML) models leveraging only clinical data are comparably effective to models incorporating additional imaging features in predicting the 2-year WOMAC score of knee osteoarthritis patients. Gradient boosting machine algorithms emerged as the top-performing ML models for this outcome during external validation, achieving clinically acceptable predictive AUC scores. In the clinical context, this suggests that patient prognosis can be successfully estimated using routinely collected patient data only, providing an opportunity to enhance patient assessment, facilitate timely interventions and avoid unnecessary imaging costs.

## Figures and Tables

**Table 1:** Final summary of the list of features used to train the machine learning algorithms.

Model	Category	Feature
Clinical and Imaging Datasets	Patient Demographics	Age
		Sex
		Ethnicity
		Living Status
		Education Status
		Employment Status
		Body Mass Index (BMI)
	Past Medical/Surgical History	Comorbidities (Charlson Comorbidity Index)
		Inflammatory Arthritis
		Injury to knee
		Knee Surgery
	Drug History	Osteoarthritis medication
		Osteoporosis medication
		Analgesic medication
	Baseline Examination	Hypertension
		20 m walk assessment
	Baseline Questionnaire	Short Form-12 (SF-12) Mental Component
		Physical Activity Scale for Elderly (PASE) score
Imaging Dataset	Radiograph	Joint Space Narrowing (JSN)—Medial
		Joint Space Narrowing (JSN)—Lateral
		Kellgren–Lawrence (KL) Grade
	Magnetic Resonance Imaging	Cartilage morphology (medial femorotibial joint)
		Cartilage morphology (lateral femorotibial joint)
		Cartilage morphology (patellofemoral joint)
		Bone marrow lesions (medial femorotibial joint)
		Bone marrow lesions (lateral femorotibial joint)
		Bone marrow lesions (patellofemoral joint)
		Meniscal tear
Outcome		2-year WOMAC score

**Table 2:** List of the baseline features and their most populated subgroup, with the total number (N) and percentage (%) of observations recorded at that level in Osteoarthritis Initiative (OAI) and Multicenter Osteoarthritis Study (MOST) databases.

Feature	Most Common Subgroup	OAI, N (%) (n = 2408)	MOST, N (%) (n = 629)
Age	60–70 years	827 (34.3)	238 (37.8)
Sex	Female	1531 (63.6)	369 (58.7)
Ethnicity	White/Caucasian	2031 (84.3)	563 (89.5)
Living Status	Lives with someone	1932 (80.2)	525 (83.5)
Education Status	Graduate degree	757 (31.4)	147 (23.4)
Employment Status	Paid work	1430 (59.4)	420 (66.8)
Body Mass Index (BMI)	Overweight (25.0–29.9)	982 (40.8)	258 (41.0)
Comorbidities (Charlson Comorbidity Index)	None	1846 (76.7)	485 (77.1)
Inflammatory Arthritis	None	2291 (95.1)	621 (98.7)
Injury to knee	None	1293 (53.7)	372 (59.1)
Knee Surgery	None	1807 (75.0)	522 (83.0)
Osteoarthritis medication	None	1480 (61.5)	434 (69.0)
Osteoporosis medication	None	1095 (45.5)	316 (50.2)
Analgesic medication	None	1453 (60.3)	154 (24.5)
Hypertension	Normal (SBP ^a^ < 140 & DBP ^a^ < 90)	1919 (79.7)	512 (81.4)
20m walk assessment	Normal pace (≥1.22 s)	1692 (70.3)	392 (62.3)
Short Form-12(SF-12) Mental Component	Normal mental health status	1214 (50.4)	319 (50.7)
Physical Activity Scale for Elderly (PASE)	Normal physical activity (≥120)	1614 (67.0)	482 (76.6)
Joint Space Narrowing (JSN)—Medial	None	974 (40.4)	391 (62.2)
Joint Space Narrowing (JSN)—Lateral	None	1905 (79.1)	509 (80.9)
Kellgren–Lawrence (KL) Grade	Moderate (KL = 3)	739 (30.7)	79 (12.6)
Cartilage morphology (medial FTJ ^b^)	No thickness loss	937 (38.9)	271 (43.1)
Cartilage morphology (lateral FTJ ^b^)	No thickness loss	1144 (47.5)	345 (54.8)
Cartilage morphology (PFJ ^b^)	Thickness loss in one or more subregion	1463 (60.8)	145 (23.1)
Bone marrow lesions (medial FTJ ^b^)	None	1532 (63.6)	474 (75.4)
Bone marrow lesions (lateral FTJ ^b^)	None	1899 (78.9)	542 (86.2)
Bone marrow lesions (PFJ ^b^)	None	940 (39.0)	283 (45.0)
Meniscal tear	None	1151 (47.8)	415 (66.0)
WOMAC	Normal (<24)	1775 (73.7)	460 (73.1)

^a^ SBP, Systolic Blood Pressure; DBP, Diastolic Blood Pressure. ^b^ FTJ, Femorotibial Joint; PFJ, Patellofemoral Joint.

**Table 3:** Area Under Curve (AUC) scores (with 95% Confidence Intervals) of six machine learning algorithms that underwent training and internal tests for clinical and imaging datasets.

ML Algorithm	Clinical Dataset		Imaging Dataset	
	Training AUC (95% CI)	Internal Test AUC (95% CI)	Training AUC (95% CI)	Internal Test AUC (95%CI)
Logistic	0.745 (0.721–0.770)	0.749 (0.700–0.797)	0.791 (0.768–0.814)	0.732 (0.682–0.782)
Lasso	0.734 (0.709–0.759)	0.751 (0.703–0.800)	0.779 (0.755–0.803)	0.738 (0.688–0.787)
Ridge	0.730 (0.705–0.756)	0.753 (0.705–0.801)	0.777 (0.753–0.801)	0.745 (0.696–0.795)
Decision Tree	0.628 (0.602–0.655)	0.630 (0.577–0.682)	0.667 (0.639–0.694)	0.654 (0.600–0.707)
Random Forest	0.784 (0.761–0.808)	0.777 (0.730–0.823)	0.820 (0.799–0.842)	0.786 (0.739–0.832)
GBM	0.736 (0.711–0.761)	0.759 (0.712–0.806)	0.783 (0.760–0.807)	0.752 (0.703–0.801)

**Table 4:** F1 scores of six machine learning algorithms that underwent internal and external tests for clinical and imaging datasets.

ML Algorithm	Clinical Dataset		Imaging Dataset	
	Internal Test F1	External Test F1	Internal Test F1	External Test F1
Logistic	0.526	0.547	0.550	0.512
Lasso	0.528	0.534	0.545	0.523
Ridge	0.536	0.541	0.543	0.522
Decision Tree	0.473	0.286	0.431	0.444
Random Forest	0.566	0.529	0.617	0.536
GBM	0.539	0.525	0.558	0.548

**Table 5:** Top five most influential features in the best performing Gradient Boosting Machine (GBM) model for clinical and imaging datasets.

Clinical Dataset	Influence Factor	Imaging Dataset	Influence Factor
Education Background	21.99	KL Grade	9.60
Arthritis History	10.56	Education Background	7.66
Comorbidities	9.73	20 m walk test	7.62
Osteoporosis medication	8.59	JSN—Medial	7.46
Past Knee Surgery	6.70	Pain medication	5.85

**Figure A1:**
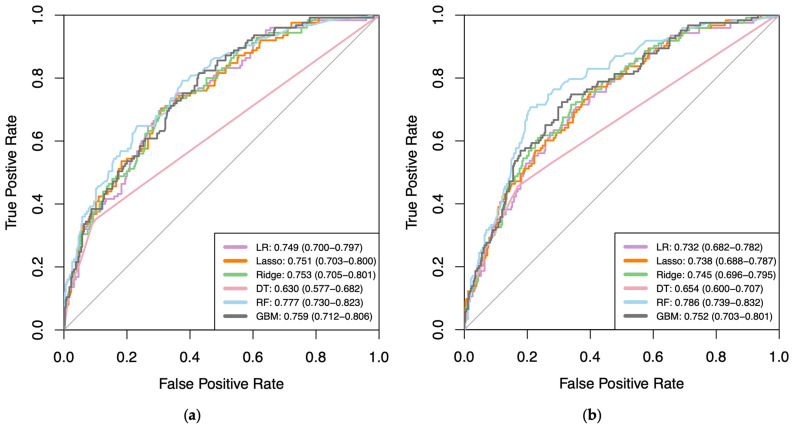
Receiver Operating Characteristic (ROC) curves showing Area Under Curve (AUC) scores (with 95% Confidence Intervals) of all six (**a**) Clinical and (**b**) Imaging Machine Learning algorithms in the Training Set from Osteoarthritis Initiative. Thin black line represents performance of a random classifier (AUC = 0.500). All values shown to 3 significant figures. LR, Logistic Regression; DT, Decision Tree; RF, Random Forest; GBM, Gradient Boosting Machine.

**Figure A2:**
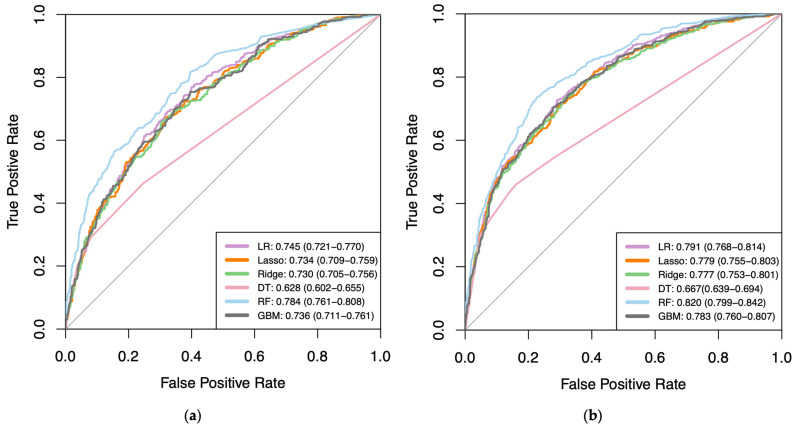
Receiver Operating Characteristic (ROC) curves showing Area Under Curve (AUC) scores (with 95% Confidence Intervals) of all six (**a**) Clinical and (**b**) Imaging Machine Learning algorithms in the Internal Test Set from Osteoarthritis Initiative. Thin black line represents performance of a random classifier (AUC = 0.500). All values shown to 3 significant figures. LR, Logistic Regression; DT, Decision Tree; RF, Random Forest; GBM, Gradient Boosting Machine.

## Data Availability

The OAI dataset is hosted by the Osteoarthritis Initiative Data Coordinating Center (OAI DCC) at the University of California, San Francisco (UCSF), and is available through the National Institute of Health (NIH) NIAMS repository: https://nda.nih.gov/oai (accessed on 5 March 2021). The MOST data are accessible through the MOST Online Data Repository and supported by the NIH NIAMS: https://most.ucsf.edu/ (accessed on 5 March 2021).

## Appendix A

**Table A1:** All subgroups of each patient feature used to train machine learning models.

Patient Feature	Subgroups within Each Feature
Age	Age ≤ 50; 50 < Age < 60; 60 ≤ Age < 70; Age ≥ 70
Sex	Male; Female
Ethnicity	White/Caucasian; Black/African American/Asian & other Non-White
Living Status	Lives Alone; Lives with someone else
Education Status	Less than high school graduate; High school graduate; Some college; College graduate; Some graduate school; Graduate degree
Employment Status	Yes; No
Body Mass Index (BMI)	Underweight (BMI < 18.5); Healthy (18.5–24.9); Overweight (25.0–29.9); Obese (30.0–39.9); Morbidly obese (BMI > 40)
Comorbidities (Charlson Comorbidity Index)	None; Mild (CCI = 1–2), Moderate (CCI = 3–4); Severe (CCI > 5)
Inflammatory Arthritis	None; OA/degenerative only; gout/other only; OA/degenerative and gout/other
Injury to knee	Yes; No
Knee Surgery	No; Left or Right; Left and Right
Osteoarthritis medication	None; corticosteroids; supplements (methylsulfonylmethane, fluorides, glucosamine); Combination of above
Osteoporosis medication	None; Vitamin D/Calcium; Bisphosphonate; Oestrogen/Raloxifene; Calcitonin/Teriparatide; Combination of above
Analgesic medication	None; WHO Pain Ladder 1 (mild); WHO Pain Ladder 2 and above (moderate to severe)
Hypertension	Normal (SBP < 140 & DBP < 90); Stage 1 (SBP ≥ 140/DBP ≥ 90); Stage 2 (SBP ≥ 160/DBP ≥ 100); Severe (SBP > 180 or DBP > 110)
20m walk assessment	No risk; Risk of disability (based on cut-off point of ≥10 s)
Short Form-12 (SF-12) Mental	normal; low mental health score
Physical Activity Scale for Elderly (PASE) score	Normal physical activity (≥120); Low physical activity (<120)
Joint Space Narrowing (JSN)—Medial	Osteoarthritis Research Society International (OARSI) Grade 0–3
Joint Space Narrowing (JSN)—Lateral	Osteoarthritis Research Society International (OARSI) Grade 0–3
Kellgren–Lawrence Grade	Normal (0); Doubtful (1); Mild (2); Moderate (3); Severe (4)
Cartilage morphology (medial femorotibial joint)	None; thickness loss in one subregion; thickness loss in more than one subregion
Cartilage morphology (lateral femorotibial joint)	None; thickness loss in one subregion; thickness loss in more than one subregion
Cartilage morphology (patellofemoral joint)	None; thickness loss in one subregion; thickness loss in more than one subregion
Bone marrow lesions (medial femorotibial joint)	None; in one subregion; in more than one subregion
Bone marrow lesions (lateral femorotibial joint)	None; in one subregion; in more than one subregion
Bone marrow lesions (patellofemoral joint)	None; in one subregion; in more than one subregion
Meniscal tear	None; in one subregion; in more than one subregion
WOMAC	WOMAC < 24; WOMAC ≥ 24

**Table A2:** Software packages used for data interpretation tasks, including data pre-processing, performance analysis and machine learning model training.

Data Interpretation Tasks	RStudio Software Package
Data Visualisation	Amelia (version 1.8.0)
Collinearity Visualisation	corrplot (version 0.92)
Data Pre-Processing—setting seed; sample split	simEd (version 2.0.0); caTools (version 1.17.1)
Area Under Curve Score; Receiver Operative Characteristic Curves	ROCR (version 1.0-11); pROC (version 1.18.0)
F1 Score—confusionMatrix	caret (version 3.45)
Generalised Linear Models (Logistic Regression)	glm (version 3.6.2)
Regularised General Linear Models (Lasso Regression)	glmnet (version 4.1-4)
Regularised General Linear Models (Ridge Regression)	glmnet (version 4.1-4)
Recursive Partitioning and Regression Trees (Decision Tree)	rpart (version 4.1.16)
Breiman and Cutler’s Random Forest Models	randomForest (version 4.7-1.1)
Generalised Boosted Regression Models	gbm (version 2.1.8)

## Appendix B

**Table A3:** Change in Area Under Curve (AUC) and F1 scores of Machine Learning models at internal and external validation, when adding imaging features to the dataset.

ML Algorithm	Internal Test		External Test	
	Change in AUC *	Change in F1 *	Change in AUC *	Change in F1 *
Logistic	−0.017	0.024	0.02	−0.035
Lasso	−0.013	0.017	0.014	−0.011
Ridge	−0.008	0.007	0.02	−0.019
Decision Tree	0.024	−0.042	−0.087	0.158
Random Forest	0.009	0.051	0.017	0.007
GBM	−0.007	0.019	0.018	0.023

* The changes in AUC and F1 scores are calculated by the difference between the scores of Imaging and Clinical Datasets.
